# Pretreatment tumor sampling and prognostic factors in patients with soft-tissue sarcoma of the head and neck

**DOI:** 10.1007/s00405-021-07162-0

**Published:** 2021-11-12

**Authors:** Johan H. Roos, Antti A. Mäkitie, Jussi Tarkkanen, Taru T. Ilmarinen

**Affiliations:** 1 Department of Otolaryngology, Head and Neck Surgery, University of Helsinki and HUS Helsinki University Hospital, Helsinki, Finland; 2 Department of Pathology, HUSLAB, University of Helsinki and Helsinki University Hospital, Helsinki, Finland; 3 Research Program in Systems Oncology, Faculty of Medicine, University of Helsinki, Helsinki, Finland

**Keywords:** Head and neck sarcoma, Prognostic factors, Pretreatment work-up, Survival, Single-center experience

## Abstract

**Background:**

Insufficient preoperative work-up and consequent intralesional or marginal resection of soft-tissue sarcomas of the head and neck (STSHNs) is common.

**Methods:**

This retrospective cohort study comprised 63 patients with STSHN treated at the Helsinki University Hospital between 2005 and 2017. We assessed the effect of pretreatment tumor sampling on surgical margin status and need for supplemental surgery, as well as prognostic factors and survival.

**Results:**

The lack of representative pretreatment biopsy specimen was associated with unfavorable margin status. Primary surgery at a non-academic center was associated with need for supplemental surgery. The 3-year overall survival (OS) was 68%, disease-specific survival (DSS) 71%, and recurrence-free survival (RFS) 61%. Higher tumor grade and primary tumor size over 5 cm were associated with reduced DSS.

**Conclusions:**

Diagnosis and management of STSHNs should be centralized to experienced academic centers. Decision-making between needle biopsy, open biopsy, or upfront radical surgery depends on tumor location and size.

**Supplementary Information:**

The online version contains supplementary material available at 10.1007/s00405-021-07162-0.

## Introduction

Soft-tissue sarcomas (STSs) develop from mesenchymal tissue and form a rare and heterogenic group of malignancies. Soft-tissue sarcomas of the head and neck (STSHNs) comprise <1% of all head and neck cancers and 5–11% of all soft-tissue sarcomas [1–3]. In Europe, age-standardized incidence of STSHN is 0.2 per 100,000 [1]. There is a slight male predominance [4–6], and the recurrence rate is higher than for STSs of other anatomic regions [7]. Five-year overall survival for STSHN (60%) is worse compared with survival for STS of the trunk and limb (80%).

Over 50 histological STS subtypes have been recognized [8]. The most frequent subtypes are angiosarcoma, fibrosarcoma, unclassified sarcoma, rhabdomyosarcoma, undifferentiated pleomorphic sarcoma (formerly known as malignant fibrous histiocytoma), leiomyosarcoma, liposarcoma, synovial sarcoma and malignant peripheral nerve sheath tumor. The most commonly used grading system is the three-tiered system by Fédération nationale des centres de lutte contre le cancer (FNCLCC).

Although certain clinical characteristics between different subtypes are shared, STSs are neoplasms with differing biological behavior. Pathologic diagnosis is difficult due to the vast array of histologic subtypes, varying grade, and similarities to other malignancies, such as carcinoma, lymphoma and melanoma [9]. Accurate diagnosis is vital for decision-making. Fortunately, diagnostics of STS has made significant strides through the evolution of immunohistochemical and molecular genetic methods.

Up until 2017, when the 8th edition of the AJCC Cancer Staging manual was published, TNM staging of STS was the same for all anatomical sites. Due to the surrounding critical anatomical structures, and more confined anatomy of the head and neck, the tumors of the head and neck tend to be smaller at diagnosis. Tumor invasion of adjacent structures affect treatment and prognosis, even though biological behavior is similar to the equivalent counterparts outside the head and neck. This was addressed in the newest edition by choosing tumor size cutoffs by traditional head and neck carcinoma size criteria [10]. The newest AJCC staging system is not recommended for angiosarcomas, rhabdomyosarcomas, rhabdoid tumors, Kaposi sarcoma, and dermatofibrosarcoma protuberans, due to dissimilarities in behavior. Important prognostic parameters, such as tumor histology, grade, and surgical resection margins are not considered in the most recent manual, and prognostic stage grouping (stage I–IV) is not applied [11].

Most patients presenting with STSHN are asymptomatic or present with a painless mass. Other symptoms include pain, nasal obstruction, epistaxis, dysphagia, proptosis, visual impairment and cranial nerve deficits [12, 13]. The most common tumor locations are superficial facial skin and scalp, neck and parotid gland, and sinonasal cavities. Nodal involvement and distant metastasis are rare at presentation.

Surgery with negative margins is considered the gold standard for treatment of STS. Negative margins in the head and neck area are difficult to achieve. Since STSHNs are typically misinterpreted as benign tumors, they are often removed without appropriate preoperative work-up. This often leads to suboptimal margins and need for supplemental surgery. Adjuvant radiotherapy is recommended in high-grade tumors and in the case of close or positive margins. However, the prognostic significance of margin status is controversial, maybe because the risk for local recurrence varies between histological STS subtypes, and the sample sizes in published reports are typically small [14]. Chemotherapy is effective against specific histological subtypes, such as pediatric rhabdomyosarcomas and Ewing family tumors, but otherwise its role is limited [9].

The aim of this study was to report on a series of STSHNs treated at the Helsinki University Hospital (HUS), and to assess factors affecting treatment outcome and survival. Special attention was paid to the value of diagnostic pretreatment tumor sampling by needle aspiration or excisional biopsy.

## Materials and methods

In this retrospective cohort study, we included both pediatric and adult patients with primary STSHN, managed at the Helsinki University Hospital (HUS, Helsinki, Finland) between 2005 and 2017. The patients were retrieved from a database held by the HUS Department of Pathology, and cross-searched from the hospital patient database using the International Classification of Diseases code C49 (malignant neoplasm of other connective and soft tissue).

We also included patients referred from other hospitals in Finland. Their treatment and follow-up had in some cases commenced or continued afterwards at the hospital close to the patient’s place of residence.

Patients with insufficient follow-up data, with primary tumors outside the head and neck, and those completely managed outside HUS were excluded. In addition, chondrosarcomas, osteosarcomas, Kaposi sarcomas, carcinosarcomas, intracerebral sarcomas and aggressive fibromatosis were excluded. Data on gender, presenting symptoms and their duration, anatomical location, biopsies, histology and immunochemistry, primary tumor grade, size and invasion, metastasis, treatment and surgical margins, recurrence, and follow-up were collected. Surgical margin status was based on the pathologist’s report. Otherwise, margins were reported as radical if they exceeded 1 cm. However, we combined radical and marginal margin status into one group in the statistical analysis. Patients with marginal and radical resections were a group with successful removal of macroscopically visible tumor. Contrarily, patients with intralesional resections were considered to have residual disease.

End points were overall survival (OS), disease-specific survival (DSS) and recurrence-free survival (RFS). OS was defined as the time interval between diagnosis and death of any cause. DSS was defined as the duration from diagnosis to death caused by STSHN. RFS was defined as the time interval between diagnosis and date of recurrence. The dates of death were provided by Statistics Finland.

The search terms used for anatomic sites were the following: soft tissue, muscle, skin, fat, maxilla, mandible, larynx, pharynx, paranasal sinuses, neck, ear, nose, oral mucosa, tongue, tonsils, parotid, submandibular gland and scalp. The search terms for histology were as follows: sarcoma, liposarcoma, rhabdomyosarcoma, sarcoma Ewing, malignant peripheral nerve sheath tumor, angiosarcoma, leiomyosarcoma, fibrosarcoma, dermatofibrosarcoma, undifferentiated pleomorphic sarcoma/malignant fibrous histiocytoma, synovial sarcoma, myoepithelioma and myxofibrosarcoma.

### Statistical analyses

All statistical analyses were calculated using IBM SPSS Statistics version 25 (IBM Corporation, Armonk, NY, USA). Chi-square and Fisher’s tests were used to compare association between two categorical variables. Differences in 3-year OS, DSS and RFS between any two groups were calculated using the Kaplan–Meier method with log rank test. *p* values under 0.05 were considered significant. Median follow-up time was calculated using the reverse Kaplan–Meier method.

## Results

### Patients

In total, 63 patients were included. The male to female ratio was 1.5 (38 males and 25 females). The average age at diagnosis was 53 years (0–89). A table presenting data on clinicopathological characteristics of all study patients is provided in the supplementary material.

### Clinical presentation

The most frequent presenting symptom was a painless mass in 33 patients (52%), followed by a skin lesion in 11 (17%). Less common symptoms were pain (14%), swelling (11%) and nasal obstruction (7.9%). Infection, epistaxis, exophthalmos and watery eyes were symptoms in a few patients. Average symptom duration was 4 months (0–12).

### Site and histology

Tumors were most commonly located in the face (23.8%), scalp (19.0%), neck (17.5%), nasal cavities (12.7%), or salivary glands (7.9%). Other locations were oral cavity, mandible, maxilla and orbit. The most frequent histological subtypes were rhabdomyosarcoma in 13, unclassified sarcoma in 11, undifferentiated pleomorphic sarcoma in nine, and angiosarcoma in eight patients. Of 63 patients, seven (11%) had neck lymph node metastases, and four (6.3%) had distant metastases at presentation.

### Pretreatment diagnosis

Needle aspiration (fine or core) was obtained in 14 of 63 (22%) patients and was suggestive of sarcoma in six of 14 (43%), and suggestive of malignancy not otherwise specified in two of 14 (14%) patients. In six of 14 (43%) patients, the material was either suggestive of non-malignant tumor or considered insufficient for diagnosis, or both. Nine of 14 patients undergoing needle aspiration had a tumor of the neck or major salivary glands, and needle aspiration was suggestive of sarcoma only in three of nine (33%) patients.

In patients undergoing open excisional biopsy, a small part of the tumor was resected for diagnostic purposes. Open biopsy was obtained in 38 of 63 (60%) and was suggestive of sarcoma in 24 of 38 (63%), and suggestive of malignancy not otherwise specified in five of 38 (13%) patients. In nine of 38 (24%) patients, the material was either suggestive of non-malignant tumor or considered insufficient for diagnosis, or both. In 17 of 39 (44%) patients undergoing open excisional biopsy, the tumor was located in the scalp or face, and sarcoma was suspected in 13 of 17 (76%) specimens. All 10 patients presenting with orbit or sinonasal tumors underwent excisional biopsy, which was suggestive of sarcoma in six of 10 (60%).

Of the 42 patients primarily treated with surgery, sarcoma diagnosis was preoperatively confirmed either by needle aspiration or by biopsy in 13 (31%). Of 29 patients without preoperative diagnosis of sarcoma, 19 (66%) were reported with intralesional surgical margins, and 12 of 29 (41%) underwent supplemental surgery. Contrarily, of 13 patients with confirmed preoperative diagnosis of sarcoma, only four (31%) presented with intralesional surgical margins, and three of 11 (27%) underwent supplemental surgery. Thus, preoperative diagnosis of sarcoma was significantly associated with favorable margin status at first surgery (*p* = 0.036). The association between preoperative sarcoma diagnosis and supplemental surgery was non-significant (*p* = 0.314).

### Treatment

#### Primary surgery

Out of the 59 patients treated with curative intent, forty (68%) were primarily treated with surgery, and 28 of these 40 (70%) were treated at HUS or another university hospital. Of the 40 patients, fourteen (35%) were reoperated because of close or positive margins. Of the 28 patients undergoing primary surgery at a university hospital, five (18%) required supplemental surgery. Of 12 patients undergoing primary surgery at a non-academic center, nine (75%) required supplemental surgery. Thus, primary surgery at a university hospital was associated with reduced need for supplemental surgery (*p* = 0.001). However, primary surgery at a university hospital was not associated with better surgical margin status (*p* = 0.5). Fourteen patients received postoperative radiotherapy and four received postoperative chemotherapy.

Primary surgical margins were intralesional in 22 and radical or marginal in 18. Surgical margin status was not statistically significantly associated with local recurrence (*p* = 0.73).

#### Primary radiotherapy/chemotherapy

**Fig. 1:**
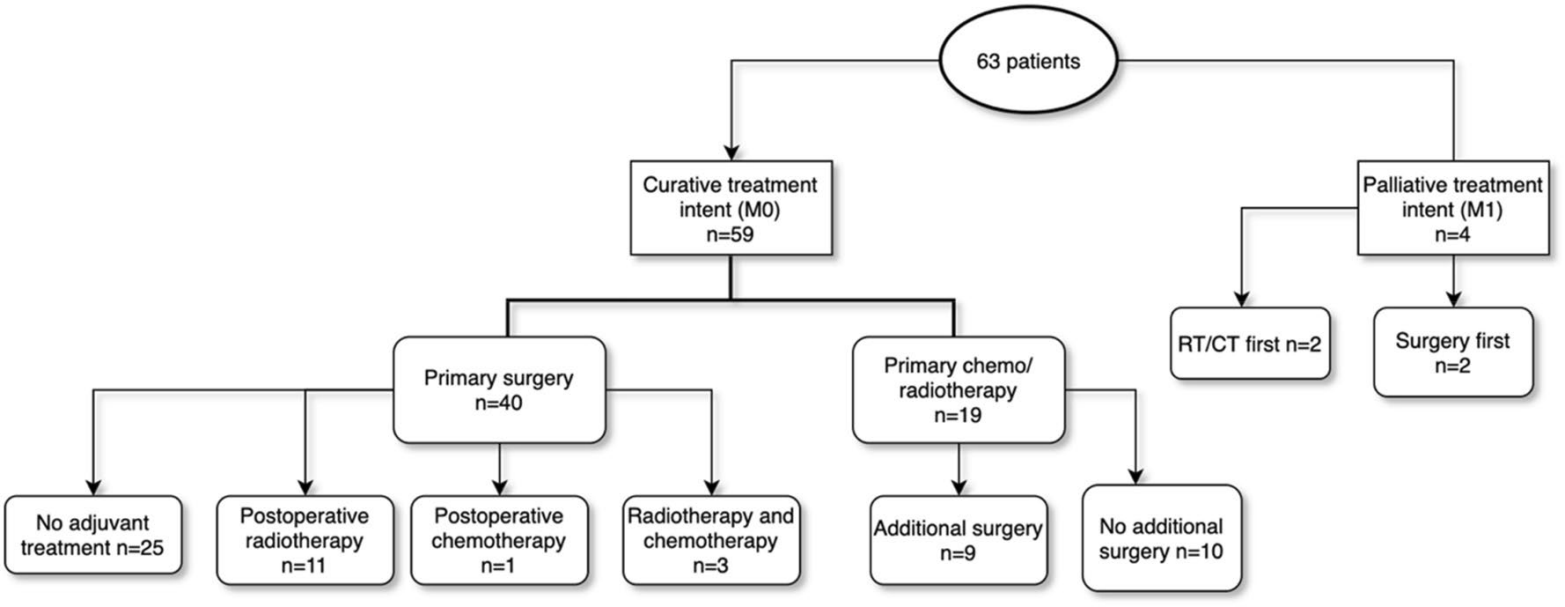
Primary treatment of 63 patients with soft-tissue sarcoma of the head and neck

Nineteen patients with curative treatment intent received primary radiotherapy and/or chemotherapy. Nine of them underwent surgery after the primary treatment. Three patients received solely radiotherapy (one declined surgery and two had angiosarcoma) (Fig. 1).

### Follow-up and outcome

The follow-up of surviving patients ranged from 6 to 128 months (median, 65). Of the 37 surviving patients, 29 (78%) had a minimum follow-up of 3 years at HUS, which was then continued elsewhere in collaboration with HUS.

Of the 59 patients primarily presenting without distant metastases, nine (15%) had persistent disease despite curative-intent treatment, and 20 (34%) had recurrent disease. The first recurrence was local in 10, regional in one, and distant in nine. Distant metastases presented mostly in the lungs. Other locations of metastasis included muscle, bone and liver.

At the end of follow-up, 36 (57%) were alive without STSHN, and one (2%) alive with STSHN. Overall, 26 of 63 (41%) patients died during follow-up. Of these 26 patients, 22 (85%) died of STSHN, and four (15%) of other reasons. The 3-year OS was 68%, DSS 71%, and RFS 61%. Table 1 presents univariate analysis of factors affecting 3-year survival in patients treated with curative intent.

**Table 1:** Univariate analysis of factors affecting 3-year overall (OS), disease-specific (DSS) and recurrence-free (RFS) survival in 59 curatively treated patients with soft-tissue sarcoma of the head and neck (Kaplan Meier with Log-rank test)

	*n*	3-year OS		3-year DSS		*n*	3-year RFS	
		(%)	*p*	(%)	*p*		(%)	*p*
Gender			0.786		0.611			0.348
Female	22	77.3		81.6		19	57.8	
Male	37	70.3		72.6		34	67.8	
Grade			**0.003**		**0.001**			0.153
Low or intermediate	22	90.9		95.5		21	82.8	
High	25	56.0		59.1		21	60.0	
First surgery			0.490		0.429			0.903
Academic center	36	69.4		71.8		32	62.6	
Non-academic center	13	84.6		84.6		12	71.4	
Age			0.129		0.208			0.157
< 20 (not included)	12					12		
20–59	17	70.6		70.6		14	52.2	
60–79	21	81.0		85.2		20	78.2	
≥ 80	10	50.0		58.3		8	66.7	
Histology			**0.042**		**0.041**			0.232
Angiosarcoma/UPS	15	60.0		72.0		13	72.9	
Rhabdomyosarcoma	12	75.0		75.0		11	34.1	
Lipo/DFSP/fibro/HPC	12	100		100		12	82.5	
Sarcoma NOS/other	20	65.0		65.0		17	68.2	
Size			0.111		0.194			0.571
≤ 2	16	81.3		81.3		15	71.4	
> 2 and ≤ 4	15	100		100		15	55.0	
> 4	23	52.2		58.7		19	71.3	
Size ≤ 5 cm	37	83.8	0.234	89.2	**0.047**	35	67.5	0.720
> 5 cm	17	52.9		52.9		14	63.5	
T class (new)			0.394		0.214			0.630
1	12	83.3		83.3		12	90.9	
2	7	85.7		85.7		6	83.3	
3	9	44.4		44.4		6	100	
4	4	75.0		75.0		3	66.7	
T class (old)			0.200		0.107			0.522
1	22	81.8		81.8		20	89.5	
2	6	33.3		33.3		4	100	
3	4	75.0		75.0		3	66.7	
N class			0.327		0.159			0.231
0	52	75.0		78.6		47	69.5	
1	7	57.1		57.1		6	33.3	
Primary surgery			0.635		0.458			0.656
Radical or marginal	24	75.0		78.9		22	69.6	
Intralesional	24	70.8		70.8		21	58.6	
Treatment			0.270		0.143			0.197
Surgery	25	84.0		84.0		22	79.6	
RT/CRT	10	70.0		78.8		9	64.8	
Combined	24	62.5		65.8		22	50.0	

*p* values marked with bold indicate statistically significant differences between groups

*Lipo* liposarcoma, *DFSP* dermatofibrosarcoma protuberans, *Fibro* fibrosarcoma, *HPC* hemangiopericytoma, *NOS* not otherwise specified, *RT* radiotherapy, *CRT* chemoradiotherapy

### Survival analysis

**Fig. 2:**
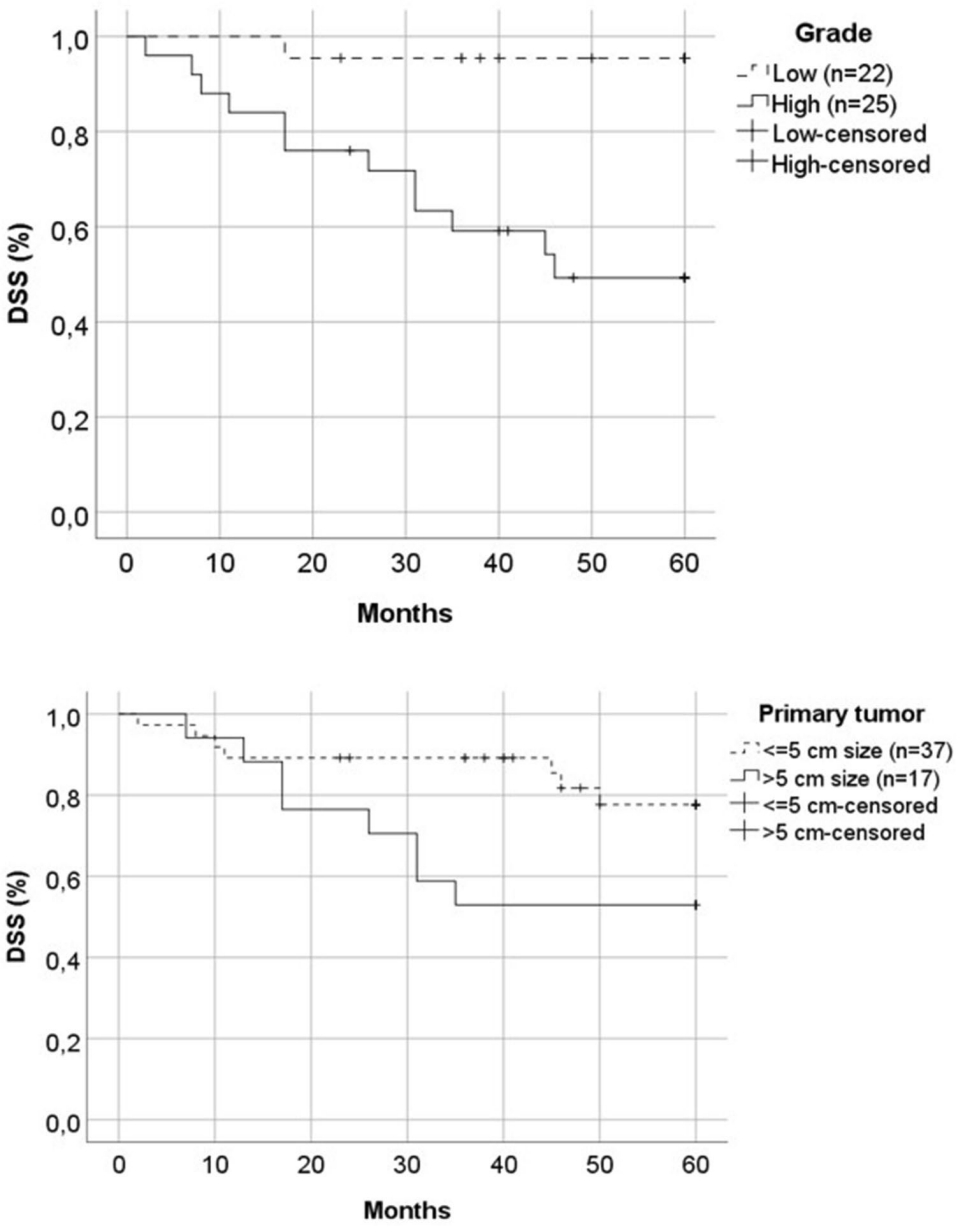
Among patients treated with curative intent, higher tumor grade (*p* = 0.001) and primary tumor size > 5 cm (*p* = 0.047) were statistically significantly associated with inferior DSS

Data on tumor differentiation (grade) were available for 47 patients primarily treated with curative intent. Higher tumor grade was statistically significantly associated with inferior DSS (*p* = 0.001) (Fig. 2).

Primary tumor size cutoffs applied by the newest AJCC staging system (≤ 2 cm versus  > 2 cm but ≤ 4 cm versus  > 4 cm) were not significantly associated with RFS (*p* = 0.571) or DSS (*p* = 0.194). However, tumors > 5 cm had inferior DSS (52.9%), compared with tumors ≤ 5 cm (*p* = 0.047). (Fig. 2).

### Comparison of histology subtypes

To assess clinical characteristics and prognosis across different histology subtypes, four groups were formed for comparison: (1) rhabdomyosarcoma, (2) angiosarcoma and undifferentiated pleomorphic sarcoma (UPS), (3) liposarcoma, dermatofibrosarcoma, fibrosarcoma and hemangiopericytoma, (4) unclassified sarcoma (sarcoma NOS) and others.

**Fig. 3:**
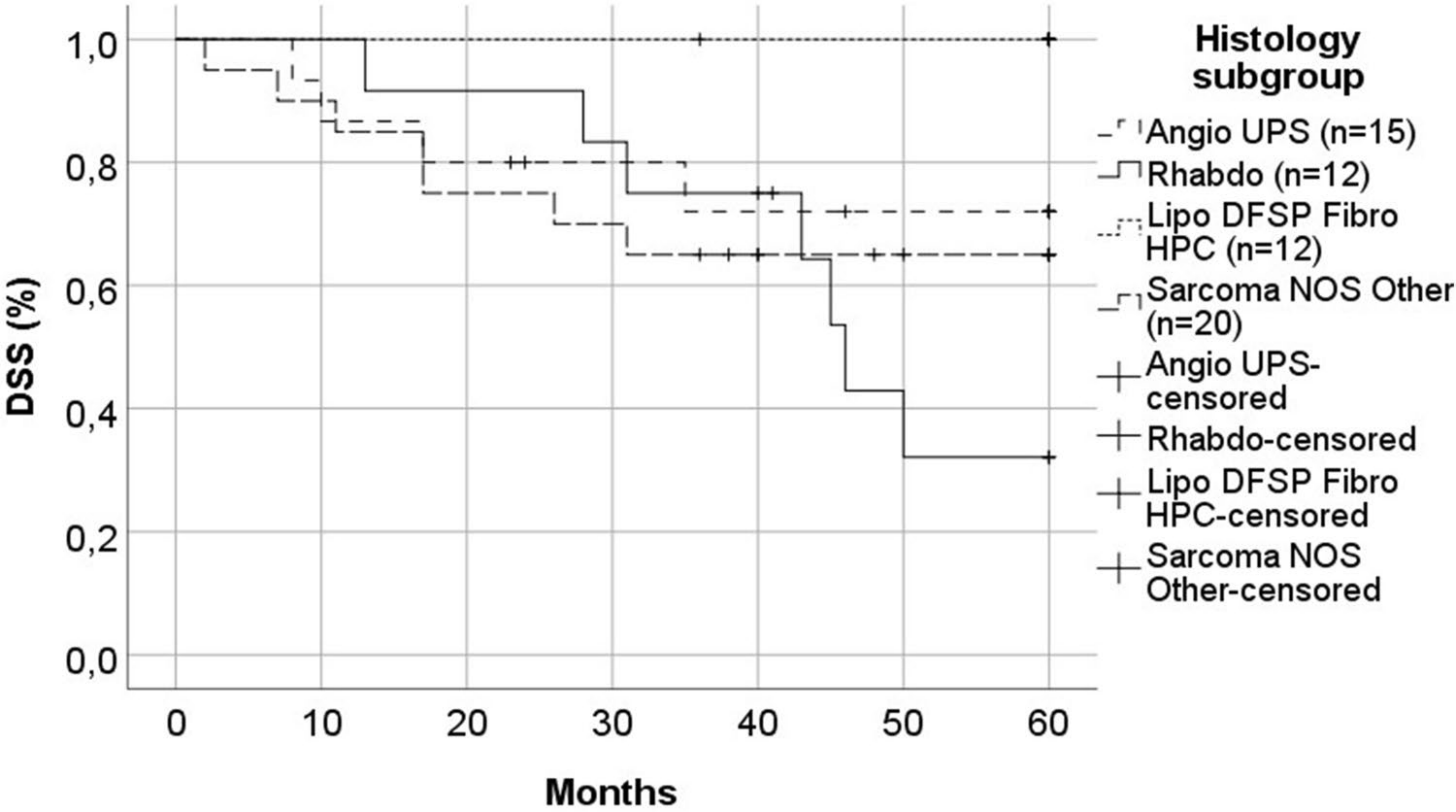
DSS of patients with rhabdomyosarcoma was statistically significantly inferior compared with patients in all other histology groups (*p* = 0.047). DSS of patients with liposarcoma, dermatofibrosarcoma protuberans (DFSP), fibrosarcoma, or hemangiopericytoma (HPC) was superior compared with other non-rhabdomyosarcomas (*p* = 0.032)

In 13 patients with rhabdomyosarcoma, the proportion of patients aged < 18 years (eight of 13, 62%) was higher compared with other histology groups, as expected. Death caused by STSHN over 3 years from diagnosis only occurred in patients with rhabdomyosarcoma, and their disease-specific survival (DSS) was inferior compared with the other histology groups (Fig. 3). Four of six rhabdomyosarcoma patients primarily treated with CRT survived, whereas only one of six patients primarily treated with surgery survived.

Of the 17 patients with angiosarcoma or undifferentiated pleomorphic sarcoma, 15 (88%) were aged over 70 years at diagnosis, and 13 of 17 (76%) presented with tumors of the face or scalp. Six out of these 17 patients (35%) died of STSHN.

Of the 13 patients with liposarcoma, dermatofibrosarcoma, fibrosarcoma or hemangiopericytoma, only three (23%) were aged over 70 years at diagnosis, and only one of 13 (8%) died of STSHN. Thus, DSS in this histology group was statistically significantly superior compared with patients with rhabdomyosarcoma, or non-rhabdomyosarcoma (Fig. 3).

## Discussion

### Pre-treatment diagnostics and planning of treatment

Since soft-tissue sarcomas are often first misinterpreted as benign tumors due to their mild symptoms and rarity, insufficient preoperative planning followed by intralesional or marginal removal is common. Our study on 63 STSHN patients suggests that the lack of representative preoperative biopsy specimen often leads to suboptimal margins. Diagnostic work-up of head and neck tumors requires expertise, and careful consideration of the surrounding anatomical structures. Whether open excisional biopsy is applicable depends on tumor location and size, and whether the tumor is covered by intact mucosa, fascia, or skin. In the present series excisional biopsy was typically obtained in tumors of the face, scalp, and orbit. When preoperative data on tumor type and grade are available, definitive treatment can be adjusted accordingly. Contrarily, if STSHN has been intralesionally removed without preoperative biopsy, determining the extent of residual tumor can be extremely difficult. Even with low-grade tumors with good prognosis, wide surgical re-resection may lead to inferior cosmetic or functional outcome. Furthermore, postoperative radiotherapy may be needed with a large target volume.

In our study, fine needle aspiration specimen was often obtained in tumors of the neck and major salivary gland. Although fine needle aspiration is the standard for evaluation of major salivary gland tumors, its value in STS is poor. Since open biopsy should be avoided in salivary gland tumors, surgical removal with careful preservation of the surrounding anatomical structures has to be accepted in certain anatomical sites, even without preoperative diagnosis.

Imaging is a vital part of the pretreatment work-up in STSHN, as it reveals important of information about the tumor size, location, and invasion into the surrounding structures. Imaging helps in defining the growth pattern of the malignancy. However, we only analyzed pathological pretreatment diagnosis in accordance with our study objectives. Histological diagnosis lays the foundation of the definitive diagnosis. Retrospective assessment of imaging studies may not be fruitful in a material representing several anatomical sites and tumor types. Furthermore, imaging protocols applied prior to treatment varied in our study patients, and some patients with small tumors did not undergo imaging prior to treatment.

Compared with patients undergoing upfront surgery at an academic center, those treated at non-academic centers were more likely to require supplemental surgery. Patients presenting with a head and neck soft-tissue tumor, not resembling any of the familiar benign lesions, should be directed to an academic center with multidisciplinary expertise in sarcoma diagnostics and treatment. Primary treatment at an academic center was not associated with favorable margin status or survival in the present study, possibly because larger, higher grade tumors and those located in challenging anatomical sites were referred to our center. A study by Gutierrez et al. showed that STS patients managed at high-volume centers have significantly better survival and functional outcomes [15].

### Comparison of results with previous similar studies

The number of patients in recent single-center studies has ranged from 36 to 186, with a mean study period of over 15 years (5–23) [3–6, 13, 16–25]. Longer study periods are typically needed to compensate for lack of statistical power. Knowledge of STS is gained with time, which should be kept in mind in regard to retrospective studies extending across longer time intervals. By implementation of novel immunohistochemical and molecular genetic methods, and additionally by improving classification of tumors, the accuracy of histological diagnosis has improved. For example, the update of WHO in 2013 modified the previous term malignant fibrous histiocytoma (MFH) into undifferentiated pleomorphic sarcoma [8]. Partly because of these reasons, we limited our study period to cover the years from 2005 to 2017.

In previous studies, the diagnosis has often changed by retrospective pathological re-evaluation of tumor histology [3, 16, 24, 26]. In our study, pathology reports and data on immunohistochemical markers were carefully reviewed by an experienced pathologist. Based on these data, the diagnoses of patients were considered accurate.

According to a review by Galy-Bernadoy et al. the six most common STSHN types are angiosarcoma, fibrosarcoma, unspecified sarcoma, rhabdomyosarcoma, malignant peripheral neural sheath tumor, and undifferentiated pleomorphic sarcoma [14]. All except MPNST were among the six most common histology types in our series. Site distribution, as well as the number of patients with lymph node and distant metastases at presentation were similar to what has been previously reported in the literature. Finland has a population of around 5.5 million people. HUS has a referral area of 1.9 M and the multidisciplinary tumor board meeting gives treatment recommendations to over one third of all head and neck cancer patients in Finland. Thus, with an incidence of 0.2 per 100 000 inhabitants, the expected number of newly diagnosed STSHNs at HUS between 2005 and 2017 is similar to what we identified by our search.

In our study, higher grade and tumor size over 5 cm were associated with an increased risk of death by STSHN. The widely adopted grading system by the French Federation of Cancer Centres (FNCLCC) was not used in our patients. Instead, division into low and high grade was used for survival analyses. In patients whose tumor grade could not be determined, OS was almost equally poor as for patients with high-grade tumors. Possibly, grading was difficult in tumors with poor differentiation. Several previous studies on STSHN have identified tumor grade as a central prognostic factor. For the purposes of upcoming studies, grading should thus be standardized. This would allow data to be accumulated for review articles to complete the 8th edition of AJCC STSHN staging. The new TNM system applies STSHN size cutoffs that head and neck specialists are familiar with i.e., 2 cm and 4 cm. This is considered applicable and practical but is not based on high-level proof of evidence. As yet, no stage grouping based on outcome is possible, and certain STS types (angiosarcoma, rhabdomyosarcoma of embryonal and alveolar subtypes, and dermatofibrosarcoma protuberans) are excluded.

According to the existing literature, the prognostic significance of margin status is one of the most important prognostic factors, but statistical significance is not reached in all studies [3, 5, 14, 17, 20, 24, 27]. However, surgery continues to be considered the gold standard of treatment of most STSHN. The risk for recurrence varies between histological STS subtypes, and the published reports typically include small patient groups with varying histologies and treatments, which could explain the lack of evidence for negative margins in some studies. In addition, in our study, other factors such as tumor grade had greater impact on survival than surgical margins. This highlights the diversity of STSHN. Perhaps future studies of specific histologic types of STSHN could closer examine margin status as a prognostic factor as opposed to chemotherapy and radiotherapy.

A previous study by Mahmoud et al. showed that adjuvant CRT seems beneficial in high-grade tumors [28]. In that study, administration of chemotherapy was not a significant predictor of survival. The outcome of childhood rhabdomyosarcoma treatment is optimized with the use of multimodality therapy [29]. Due to heterogeneity of tumors, and small number of patients with high-grade STS, conclusions from our data cannot be drawn regarding the role of oncological adjuvant treatments in STSHN.

DSS (71%), and OS (68%) in our study were similar to those reported in the previous literature. In concordance with the previous reports, DSS in patients with angiosarcoma or rhabdomyosarcoma was poor. Contrarily, prognosis of patients with liposarcoma, fibrosarcoma, DFSP, or HPC was good [3, 6, 28].

In our study, angiosarcomas mostly presented in elderly patients, typically in face or scalp. In a recent study by Smrke et al., the median age of patients with angiosarcoma was 72 years [30]. Exposure to UV radiation seems to be a risk factor for angiosarcoma. A study by Woods et al. investigating patterns of Australian STSHN subtypes, suggested ultraviolet radiation to be an epidemiological factor of UPS. This seems to fit our data, where UPS often presented in the face and scalp in older patients [31]. Age at presentation should be considered in studies comparing OS between STS histology groups.

## Conclusions

We confirmed previous findings regarding grade, size and histology being primary prognostic factors of STSHN. Decision between needle biopsy and open biopsy is dependent on the anatomic location of the malignancy, but open biopsy should be preferred when possible for a more accurate pre-treatment diagnosis. Accurate diagnosis more often leads to successful surgery with adequate margins. However, due to the small sample size in our study, this needs to be examined further to confirm the statement. Sarcoma diagnosis and treatment should be centralized to experienced multidisciplinary academic centers. Survival data in STSHN can be affected by small sample size, and study group heterogeneity. This highlights the need for meta-analyses or combined cancer databases. Standardized classification, grading and staging systems are of utmost importance to compare and combine available data.

## Supplementary Information

Below is the link to the electronic supplementary material.

Supplementary file 1 (DOCX)
