# Correction to: Pretreatment tumor sampling and prognostic factors in patients with soft-tissue sarcoma of the head and neck

**DOI:** 10.1007/s00405-021-07205-6

**Published:** 2021-12-04

**Authors:** Johan H. Roos, Antti A. Mäkitie, Jussi Tarkkanen, Taru T. Ilmarinen

**Affiliations:** 1 Department of Otolaryngology, Head and Neck Surgery, University of Helsinki and HUS Helsinki University Hospital, Helsinki, Finland; 2 Department of Pathology, HUSLAB, University of Helsinki and Helsinki University Hospital, Helsinki, Finland; 3 Research Program in Systems Oncology, Faculty of Medicine, University of Helsinki, Helsinki, Finland

## Correction to: European Archives of Oto-Rhino-Laryngology 10.1007/s00405-021-07162-0

In the original publication of the article, affiliation 1 was published incorrectly and the correct affiliation 1 should read as below,

Department of Otolaryngology, Head and Neck Surgery, University of Helsinki and HUS Helsinki University Hospital, Helsinki, Finland

In addition, under funding section, the university name should read as “Helsinki University Hospital” instead “Helsinki University Central Hospital”.

The original article was updated.

