# Supplementary material for: Pretreatment tumor sampling and prognostic factors in patients with soft-tissue sarcoma of the head and neck
Source: Eur Arch Otorhinolaryngol. 2021 Nov 12;279(6):3147–55. doi: 10.1007/s00405-021-07162-0 (PMC9072459; doi:10.1007/s00405-021-07162-0)
Supplement: Supplementary file 1 — (DOCX) [file 405_2021_7162_MOESM1_ESM.docx]

**Supplementary material: Table 1 – Patient data**

| Patient | Age, gender | Site | Size (cm) | Histology | Primary treatment | Adjuvant treatment | Recurrence | Outcome |
| --- | --- | --- | --- | --- | --- | --- | --- | --- |
| P1 | 21, F | Salivary gland | 2 | Rhabdomyosarcoma | Surgery | CRT | Yes | DOD |
| P2 | 15, M | Orbit | 3.5 | Rhabdomyosarcoma | CRT | None | Yes | ANED |
| P3 | 4, M | Orbit | 3 | Rhabdomyosarcoma | CRT | None | No | ANED |
| P4 | 17, F | Oral cavity | 9 | Rhabdomyosarcoma | Surgery | CRT | No | DOD |
| P5 | 53, F | Neck | 9 | Rhabdomyosarcoma | Surgery | CRT | NA | DOD |
| P6 | 62, M | Neck | 4 | Rhabdomyosarcoma | Surgery | CRT | Yes | DOD |
| P7 | 14, M | Sinonasal | 7 | Rhabdomyosarcoma | CRT | None | No | ANED |
| P8 | 40, M | Sinonasal | NA | Rhabdomyosarcoma | CRT | None | Yes | DOD |
| P9 | 6, F | Sinonasal | 6.9 | Rhabdomyosarcoma | CRT | None | No | ANED |
| P10 | 3, M | Face | 2.5 | Rhabdomyosarcoma | Surgery | CRT | No | ANED |
| P11 | 11, F | PPS | 4 | Rhabdomyosarcoma | Surgery | CRT | Yes | DOD |
| P12 | 46, M | Sinonasal | 7 | Rhabdomyosarcoma | CRT | None | Yes | DOD |
| P13 | 0, M | Face | NA | Rhabdomyosarcoma | Surgery | CT | Yes | DOD |
| P14 | 65, M | Face | 1 | Sarcoma NOS | Surgery | None | No | ANED |
| P15 | 37, M | Salivary gland | 3.5 | Sarcoma NOS | Surgery | None | No | ANED |
| P16 | 26, F | Mandible | 2 | Sarcoma NOS | Surgery | CRT | Yes | DOD |
| P17 | 84, F | Face | 0.4 | Sarcoma NOS | Surgery | None | No | DOC |
| P18 | 71, F | Mastoid | NA | Sarcoma NOS | Surgery | RT | No | ANED |
| P19 | 89, M | Face | 2.3 | Sarcoma NOS | Surgery | None | No | ANED |
| P20 | 62, M | Salivary gland | 5.5 | Sarcoma NOS | Surgery | RT | No | ANED |
| P21 | 64, F | Sinonasal | 3 | Sarcoma NOS | Surgery | None | Yes | ANED |
| P22 | 48, M | Neck | 10 | Sarcoma NOS | Surgery | CRT | No | DOD |
| P23 | 77, M | Maxilla | 5 | Sarcoma NOS | Surgery | None | No | DOD |
| P24 | 79, M | Neck | 6.5 | Sarcoma NOS | Surgery | None | Yes | DOD |
| P25 | 81, F | Face | 0.4 | UPS | Surgery | None | No | ANED |
| P26 | 51, M | Sinonasal | 5 | UPS | Surgery | RT | No | ANED |
| P27 | 82, M | Scalp | 4 & 2.5 | UPS | Surgery | NA | No | DOD |
| P28 | 77, M | Face | 1.5 | UPS | Surgery | None | No | ANED |
| P29 | 70, F | Salivary gland | 3.5 | UPS | Surgery | RT | Yes | ANED |
| P30 | 82, F | Oral cavity | NA | UPS | Surgery | RT | Yes | DOD |
| P31 | 80, M | Scalp | 1.5 | UPS | Surgery | None | Yes | DOD |
| P32 | 73, F | Face | 1.5 | UPS | Surgery | None | No | ANED |
| P33 | 74, M | Face | 1 | UPS | Surgery | None | No | DOC |
| P34 | 81, F | Scalp | 5 | Angiosarcoma | Surgery | RT | No | DOC |
| P35 | 83, M | Scalp | 6.3 | Angiosarcoma | Surgery | None | Yes | ANED |
| P36 | 52, F | Tongue | 1.5 | Angiosarcoma | Surgery | None | Yes | AWD |
| P37 | 81, M | Face | 8 | Angiosarcoma | RT | None | No | ANED |
| P38 | 82, F | Scalp | NA | Angiosarcoma | Surgery | CRT | No | DOD |
| P39 | 70, M | Face | 4.5 | Angiosarcoma | RT | None | No | DOC |
| P40 | 72, M | Scalp | 9 | Angiosarcoma | Surgery | CT | Yes | DOD |
| P41 | 83, F | Scalp | 3 | Angiosarcoma | Surgery | None | Yes | DOD |
| P42 | 70, F | Neck | 1.9 | Liposarcoma | Surgery | None | No | ANED |
| P43 | 65, M | Neck | 6.5 | Liposarcoma | Surgery | None | No | ANED |
| P44 | 32, M | Neck | 1.9 | Liposarcoma | Surgery | None | No | ANED |
| P45 | 40, F | Neck | 2.7 | Liposarcoma | Surgery | None | Yes | ANED |
| P46 | 47, M | Scalp | NA | Fibrosarcoma | Surgery | RT | No | ANED |
| P47 | 64, M | Scalp | 7 | Fibrosarcoma | Surgery | None | No | ANED |
| P48 | 61, M | Sinonasal | 6 | Fibrosarcoma | Surgery | RT | No | ANED |
| P49 | 74, F | Sinonasal | 6 | Fibrosarcoma | Surgery | RT | No | DOD |
| P50 | 50, M | Scalp | 2.4 | DFSP | Surgery | None | No | ANED |
| P51 | 73, F | Face | 6 | DFSP | Surgery | None | No | ANED |
| P52 | 17, M | Scalp | 3 | DFSP | Surgery | None | No | ANED |
| P53 | 65, M | Face | 1 | DFSP | Surgery | None | No | ANED |
| P54 | 38, F | Pharynx | 5 | Ewing sarcoma | Surgery | CRT | No | ANED |
| P55 | 30, M | Oral cavity | 3.3 | Ewing sarcoma | CRT | None | No | ANED |
| P56 | 3, M | Skull | 8 | Ewing-like sarcoma | Surgery | CRT | Yes | DOD |
| P57 | 79, M | Scalp | 1.6 | Myxofibrosarcoma | Surgery | None | No | ANED |
| P58 | 71, F | Face | 2 | Myxofibrosarcoma | Surgery | RT | No | ANED |
| P59 | 89, M | Neck | 7 | Myxofibrosarcoma | None | RT | No | DOD |
| P60 | 42, F | Neck | 2.5 | Synovial sarcoma | Surgery | RT | No | ANED |
| P61 | 28, M | Neck | 8 | MPNST | Surgery | None | No | DOD |
| P62 | 5, M | Salivary gland | 6 | MRT | Surgery | CRT | No | ANED |
| P63 | 15, F | Face | 4 | HPC | Surgery | RT | Yes | ANED |

Abbreviations: RT=radiotherapy, CRT=chemoradiotherapy, DFSP=dermatofibrosarcoma protuberans, UPS=undifferentiated pleomorphic sarcoma, NOS=not otherwise specified, MPNST=malignant peripheral nerve sheath tumor, MRT= malign rhabdoid tumor, HPC = hemangiopericytoma, PPS=Parapharyngeal space, CT = chemotherapy, AWD = alive with disease, ANED = alive, no evidence of disease, DOD = dead of disease, DOC = dead of other cause

a
